# The Long-Term Impact of Lorazepam on Catatonia Recurrence in Patients With Bipolar Disorder

**DOI:** 10.7759/cureus.79616

**Published:** 2025-02-25

**Authors:** Stephen Chien, Zachary Zook, Lily Charron, Eduardo D Espiridion

**Affiliations:** 1 Psychiatry, Drexel University College of Medicine, West Reading, USA; 2 Psychiatry, Drexel University College of Medicine, Philadelphia, USA

**Keywords:** bipolar disorders, medication therapy management, psychiatric symptoms, recurrent catatonia, schizophrenia and other psychotic disorders

## Abstract

Introduction: Catatonia is a complex syndrome characterized by behavioral and psychomotor abnormalities and is commonly associated with various medical and psychiatric conditions including schizophrenia, bipolar disorder, depression, stroke, and encephalitis. Lorazepam is widely considered the first-line treatment for catatonia, but the drug’s effectiveness in preventing recurrence remains unclear. This study examines whether lorazepam reduces the risk of recurrent catatonia in patients with bipolar disorder.

Methods: We conducted a retrospective cohort study using the TriNetX database, including patients who were diagnosed with bipolar disorder and catatonia between 2000 and 2024. Patients were divided into two groups: those treated with lorazepam (n = 15,674) and those who did not receive lorazepam (n = 12,045). Kaplan-Meier survival analysis and hazard ratios were used to assess recurrence risk and long-term outcomes.

Results: Overall, the catatonia recurrence rate was similar between groups (p = 0.086). However, the group that did not receive lorazepam had a statistically significantly fewer total number of recurrent episodes of catatonia than the lorazepam treatment group (p = 0.005). Additionally, Kaplan-Meier analysis showed a significantly higher survival probability in the no lorazepam group (p = 0.011), indicating a longer duration of time before recurrence in this cohort.

Conclusion: Lorazepam effectively treats catatonia acutely but does not significantly reduce the risk of recurrence compared to other management options. Future studies should explore individualized treatment approaches to optimize catatonia management for patients with bipolar disorder.

## Introduction

Catatonia is a complex web of behavioral and psychomotor symptoms that can be difficult to identify and treat. Originally a subtype of schizophrenia, catatonia is now recognized in other psychiatric disorders [1]. The Diagnostic and Statistical Manual (DSM)-5 now defines a catatonia diagnosis as the presence of at least three of 12 symptoms that include catalepsy, waxy flexibility, stupor, and agitation [1]. Immobility and mutism are particularly common clinical signs [2]. Catatonia is also used as a specifier for several disorders including schizophrenia, schizoaffective disorder, major depressive disorder, and bipolar disorder. This condition carries a prevalence of 10%-25% in those with an acute psychiatric condition [3]. Additionally, there can be a medical condition underlying catatonia, such as autoimmune encephalitis, systemic lupus erythematosus (SLE), and viral or bacterial meningitis/encephalitis [4,5]. Catatonia is an important condition to diagnose and treat because it can be concurrently associated with complications like aspiration, dehydration, venous thromboembolism, infection, pressure sores, malnutrition, and acute renal failure [4,6]. Although not defined in the DSM-5, there are generally three contexts in which catatonia is observed: akinetic catatonia, excited catatonia, and malignant catatonia [7]. As the name suggests, malignant catatonia is the most concerning and is often associated with autonomic instability and neuroleptic malignant syndrome (NMS) [8]. 

Because there is so much variation in the presentation of catatonia, diagnosis and treatment can be difficult. Other than meeting the DSM-5 requirements by observation, there is no gold standard biomarker test for catatonia [8]. In a circular pattern of reasoning, many diagnose catatonia based on a successful response to benzodiazepines, which are often the first line of treatment. This is called the lorazepam challenge, so-called by the most commonly used benzodiazepine [7,9]. Intravenous lorazepam is favored due to its ease of administration, rapid onset, and prolonged duration of action [6]. Catatonic symptoms are believed to be due to reduced gamma-aminobutyric acid (GABA) activity in the orbitofrontal and parietal cortices, hence why benzodiazepines are effective [7,8]. After lorazepam, the most common second line of treatment for catatonia is electroconvulsive therapy (ECT) [6]. ECT for catatonia involves mild electrical stimulation to the orbitofrontal and parietal cortices under anesthesia [7]. Due to the invasiveness of this procedure, it is usually only used if lorazepam treatment is ineffective within 48-72 hours or in the presence of catatonia due to NMS [6]. 

There are a few other treatments that have been explored for the management of catatonia. In addition to the changes in GABA activity, patients with catatonia have also been found to have decreases in N-methyl-D-aspartate (NMDA) (glutamate) and D2 (dopamine) receptor activity [7,10]. As a result, some atypical antipsychotics have been used to target this system [11]. Research on the efficacy of antipsychotics on catatonia can be difficult because the patients on whom antipsychotics are most effective are those with catatonia due to an underlying psychotic disorder. 

## Materials and methods

We gathered a cohort of patients across multiple healthcare organizations (HCOs) who were reported to be diagnosed with bipolar disorder presenting with catatonia. We then divided this cohort into two study groups: one treated with lorazepam (Ativan) and one without lorazepam. The retrospective cohort was obtained through the TriNetX database. TriNetX is a global health research network providing researchers access to extensive de-identified patient information extracted from the electronic health records (EHRs) of over 250 million patients worldwide. These records are sourced from more than 220 HCOs and categorized into subnetworks based on region, capabilities, and data sources. Participating HCOs, primarily academic medical centers, cover a range of healthcare encounters, including emergency department visits, outpatient care, and inpatient services. Our study focused on a network of 61 HCOs, encompassing over 105 million patients exclusively from the United States. These are the total number of HCOs that participated in TriNetX that contributed the de-identified patient data to research networks.

We included patients between the ages of 18 and 90 years and records reported from 2000 to 2024. We identified our two cohorts using inclusion and exclusion criteria from the International Classification of Diseases, 10th Revision (ICD-10) codes. All patients were required to have ICD-10-CM R29.818 other symptoms and signs affecting the nervous system-catatonia and ICD-10-CM F31 bipolar disorder. Those who were given lorazepam, defined by RxNorm 6470 in TriNetX, were included in the lorazepam group. Those who did not receive lorazepam were included in the no-lorazepam group. 

Using the analysis programming provided by TriNetX, a demographic and outcomes analyses were performed for both treatment groups. The results of the analysis included data on follow-up timelines, measures of association, a Kaplan-Meier analysis, and the number of instances. 

Given the use of de-identified patient records and the absence of any collection, use, or transmission of individually identifiable data in this retrospective cohort study, it was deemed exempt from institutional review board approval and informed consent as per the Health Insurance Portability and Accountability Act (HIPAA). Furthermore, we strictly adhered to the reporting guidelines outlined in the Strengthening the Reporting of Observational Studies in Epidemiology (STROBE) framework throughout all phases of our investigation.

## Results

The US collaborative network TriNetX has 105,703,806 patients across 61 HCOs. We were able to identify 15,674 patients in the lorazepam group and 12,045 patients in the no-lorazepam group.

Demographics were similar among both cohorts (Table 1). The lorazepam group ages at the time of their catatonia episode were M = 55.7 and SD = 16.0, while the no-lorazepam group were M = 55.6 and SD = 17.2. 61.27% (9,603) of patients in the lorazepam group were female and 32.73% (5,130) were male, while 59.14% (7,123) of the no-lorazepam group were female and 31.76% (3,826) were male. In the lorazepam group, 5.44% (852) of patients identified as Hispanic or Latino, and 68.24% (10,696) were not Hispanic or Latino. In the no-lorazepam group, 5.71% (688) patients identified as Hispanic or Latino, and 68.07% (8,199) were not Hispanic or Latino. Finally, reported races in the lorazepam group were 68.36% (10,715) White, 13.30% (2,085) Black or African-American, 1.10% (173) Asian, and 3.09% (484) Other. The races for the no-lorazepam group were 63.02% (7,591) White, 16.12% (1,941) Black, 0.87% (105) Asian, and 5.42% (653) Other.

**Table 1:** Demographics

	Lorazepam (n = 15,674)	No lorazepam (n = 12,045)	p-value
Age, mean ± SD	55.7 ± 16.0	55.6 ± 17.2	0.618
Gender			
Male, n (%)	5130 (32.7%)	3826 (31.8%)	0.091
Female n (%)	9603 (61.3%)	7123 (59.1%)	<0.001
Unknown n (%)	941 (6%)	1096 (9.1%)	<0.001
Race			
White	10715 (68.4%)	7591 (63.0%)	<0.001
Black	2085 (13.3%)	1941 (16.1%)	<0.001
Asian	173 (1.1%)	105 (0.9%)	0.063
Other	484 (3.1%)	653 (5.4%)	<0.001
Unknown, n (%)	2217 (14.1%)	1755 (14.6%)	0.324
Demographics			
Hispanic or Latino	852 (5.4%)	688 (5.7%)	0.333
Not Hispanic or Latino	10696 (68.2%)	8199 (68.1%)	0.772
Unknown, n (%)	4126 (26.3%)	3158 (26.2%)	0.854

**Figure 1:**
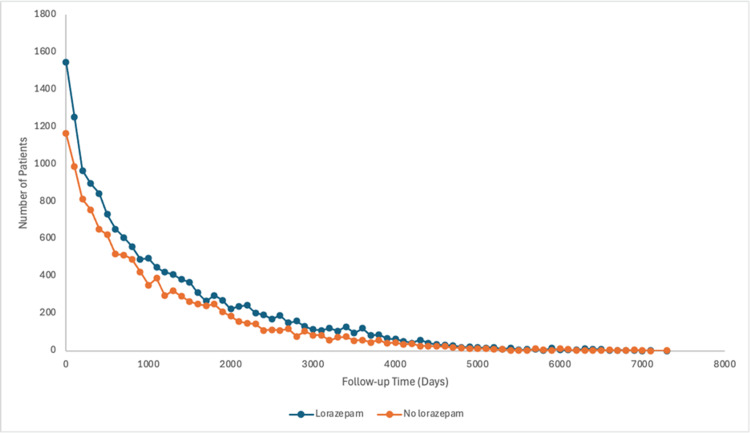
Follow-up time (days) after initial catatonic episode to measured outcome

We measured the number of follow-up days from the index event (catatonia with or without lorazepam treatment) to the outcome measured (presence or absence of another catatonic episode) (Figure 1).

In the lorazepam group, the number of follow-up days was M = 1197.07 (~3 years), SD = 1194.07, with a median of 813 days and interquartile range of 1,524 days. In the no-lorazepam group, the number of follow-up days was M = 1111.24 (~3 years), SD = 1131.60, with a median of 750 days and an interquartile range of 1,369 days (Table 2). 

**Table 2:** Follow-up time (days) after initial catatonic episode to measure outcome

Cohort name	Mean follow-up (days)	Standard deviation	Median follow-up (days)	Interquartile range
Lorazepam	1197.07	1194.07	813	1524
No lorazepam	1111.24	1131.60	750	1369

We calculated the risk and odds in each treatment cohort for another episode of catatonia after the index catatonic episode. Of the 15,674 patients in the lorazepam group, 3,962 patients had another episode of catatonia, with a risk of 0.25. In the no-lorazepam group, 3,154 of the 12,045 patients in the cohort reported another catatonic episode (Table 3).

**Table 3:** Measures of association: cohort statistics

Cohort	Patients in cohort	Patients with outcome	Risk
Lorazepam	15674	3962	0.25
No lorazepam	12045	3154	0.26

The difference in risk was not significant, z = -1.71, p = 0.086, 95% CI = (-0.019, 0.001) (Table 4).

**Table 4:** Measures of association: risk difference

Risk difference	95% CI lower	95% CI upper	z	p
-0.01	-0.019	0.001	-1.71	.086

Both the risk and odds ratio reflect this result, with the risk ratio being 0.97, 95% CI = (0.927, 1.005) and the odds ratio being 0.95, 95% CI = (0.903, 1.007) (Tables 5, 6). 

**Table 5:** Measures of association: risk ratio

Risk ratio	95% CI lower	95% CI upper
0.97	0.927	1.005

**Table 6:** Measures of association: odds ratio

Odds ratio	95% CI lower	95% CI upper
0.95	0.903	1.007

A Kaplan-Meier analysis calculated the expected survival and hazard ratios for each treatment group. The survival probability for the lorazepam group was 0.59, while the probability for the no-lorazepam group was 0.62 (Table 7).

**Table 7:** Kaplan-Meier analysis: cohort statistics

Cohort	Patients in cohort	Patients with outcome	Survival probability at end of time window
Lorazepam	15674	3962	0.59
No lorazepam	12045	3154	0.62

The log-rank test showed this difference to be statistically significant, χ²(1) = 6.39, p = .011, with the no-lorazepam group having a significantly higher expected survival rate than the lorazepam group (Table 8).

**Table 8:** Kaplan-Meier analysis: log-rank test χ²: Chi-square; df: degrees of freedom; p: p-value

χ²	df	p
6.39	1	.011

The Kaplan-Meier analysis also calculated a hazard ratio of 0.94, which was found to be statistically significant using a proportionality test, χ²(1) = 27.99, p < .0001 (Tables 9, 10). This result indicates that the no-lorazepam group was less likely to have an additional catatonic episode after the index event. 

**Table 9:** Kaplan-Meier analysis: hazard ratio

Hazard ratio	95% CI lower	95% CI upper
0.94	0.899	0.987

**Table 10:** Kaplan-Meier analysis: proportionality χ²: Chi-square; df: degrees of freedom; p: p-value

χ²	df	p
27.99	1	< .0001

Finally, we calculated the number of instances of additional catatonic events for those who reported at least one additional episode. In the lorazepam group, 3,962 patients had at least one instance of catatonia after the index event and treatment, with the number of instances being M = 3.59, SD = 8.18. The no-lorazepam group had 3,154 patients with at least 1one instance, with the number of instances being M = 3.10, SD = 6.38 (Table 11). A t-test found this to be a statistically significant difference, t = (7114) =2.80, p = .005, with the no-lorazepam group having a significantly fewer number of instances of catatonia than the lorazepam treatment group (Table 12).

**Table 11:** Number of instances: cohort statistics

Cohort	Patients in cohort	Patients with outcome	Mean	Standard deviation	Median
Lorazepam	15674	3962	3.59	8.18	1
No lorazepam	12045	3154	3.10	6.38	1

**Table 12:** Number of instances: test statistics df: degrees of freedom; p: p-value

t	df	p
2.80	7114	.005

## Discussion

This study examines the efficacy of lorazepam in the management of catatonia among patients with bipolar disorder. While effective in acute symptom resolution, the use of lorazepam did not demonstrate a statistically significant reduction in the recurrence of catatonia compared to those who did not receive lorazepam. 

One of the most notable findings was that those who did not receive lorazepam had a statistically significantly higher survival probability of avoiding catatonia recurrence (p = 0.011). In addition, those who did not receive lorazepam experienced fewer instances of catatonia, with a statistically significant difference (p = 0.005). These findings raise questions about the long-term efficacy of lorazepam in mitigating catatonia recurrence. These results may stem from differences in patient characteristics or alternative treatment strategies. Patients may have also had a milder initial presentation which influenced recurrence outcomes. 

Additionally, the observed results can also be due to the complexity of different neurotransmitter systems that are involved in catatonia. Lorazepam is a benzodiazepine that targets GABA-A receptors, but NMDA and dopamine receptors could also factor into the pathophysiology of catatonia [7,8]. This aligns with prior studies suggesting the utility of multimodal approaches, such as ECT or adjunctive antipsychotics [8]. 

Despite providing significant findings, this study has limitations that warrant consideration when interpreting the results. Firstly, the retrospective design limits the ability to establish causal relationships between lorazepam treatment and long-term catatonia outcomes. This study relies on data from the TriNetX database, which constrains the study to varying degrees of quality and completeness of EHRs, due to the different participating HCOs. 

Secondly, using ICD-10 diagnostic codes to identify catatonia may not fully capture the complexity as well as the variability of this condition. ICD-10 codes lack the specificity necessary to differentiate subtypes of catatonia, such as excited and akinetic [7]. Moreover, this study only examined catatonia in patients concurrently diagnosed with bipolar disorder. There are numerous etiologies of catatonia from a wide variety of medical and psychiatric conditions [8]. Additionally, patients with mild catatonic symptoms may not have received formal diagnoses, which leads to potential underreporting and an incomplete picture of the patient population. 

Lastly, there can be variability in the administration of lorazepam across different healthcare settings. Differences in dosing protocols and timing are not captured in our data but can have an impact on recurrence outcomes. The duration as to how long patients who were on lorazepam was not captured either. Once catatonia symptoms are remitted, lorazepam is tapered, but this can cause symptoms to return, requiring the need to continue the benzodiazepine for an unknown amount of time [11]. Furthermore, information on adjunctive therapies like ECT or other psychotropic medications, was not further delineated, which limits our ability to evaluate the role of lorazepam in catatonia management. Other benzodiazepines were not delineated or queried. One related limitation is the lack of detailed information on the treatment administered in the no-lorazepam group. Some of the more common alternatives include NMDA receptor antagonists, dopamine agonists, anticonvulsants, and antipsychotics [9,10]. Further studies should aim to gather more specific data on alternative pharmacological agents to better comprehend their roles in catatonia management and recurrence prevention.

## Conclusions

This study discusses the role of lorazepam in the treatment of catatonia in patients with bipolar disorder. Despite being a widely accepted first-line treatment, lorazepam may not significantly decrease long-term recurrence risk. The findings stress the importance of developing comprehensive treatment strategies that are individually tailored. Further research, especially randomized controlled trials, is crucial to optimize the management of this complex condition.
